# Understanding Oral Hygiene and Periodontal Health in Space Using a Bedrest Analog Model for Weightlessness (NASA Sensorimotor Countermeasures Study)

**DOI:** 10.1002/cre2.70412

**Published:** 2026-07-16

**Authors:** Sonja Henny Maria Derman, Laura de Boni, Edwin Mulder, Stefan Möstl, Julia Jagel, Teresa Kruse, Anna Greta Barbe

**Affiliations:** ^1^ Faculty of Medicine and University Hospital Cologne, Polyclinic for Operative Dentistry and Periodontology, University of Cologne Cologne Germany; ^2^ German Aerospace Center (DLR), Institute of Aerospace Medicine Cologne Germany; ^3^ Institute of Neuropathology University Hospital Bonn Bonn Germany; ^4^ Dementia Research Institute (DRI) University College London London UK; ^5^ Faculty of Medicine and University Hospital Cologne, Department of Orthodontics University of Cologne Cologne Germany

**Keywords:** dental plaque, gingivitis, microgravity, periodontal diseases, saliva, weightlessness

## Abstract

**Aims:**

To investigate longitudinal alterations in oral hygiene, gingival inflammation, and salivary secretion during a 60‐day head‐down tilt bed rest (HDTBR) protocol as a validated ground‐based analog of microgravity.

**Materials and Methods:**

Twenty‐four healthy volunteers participated in a 60‐day, 6° HDTBR study conducted under strictly controlled conditions. Oral health assessment safety measures were performed at baseline, mid‐head‐down tilt (HDT), end‐HDT, and post‐recovery.

**Results:**

Plaque accumulation and gingival inflammation increased, with increases from baseline to end‐HDT in the Quigley‐Hein Plaque Index (1.8 ± 0.4 to 2.3 ± 0.4; *p* < 0.01), Gingival Index (0.8 ± 0.4 to 1.2 ± 0.2; *p* < 0.001), and bleeding on probing (BoP) (18.4 ± 11.6% to 35.1 ± 16.6%; *p* < 0.001). BoP partially recovered post‐HDT, while QHI and GI remained elevated compared with baseline. Periodontal Screening Index scores shifted toward higher grades during HDT without evidence of destructive periodontal breakdown. The unstimulated salivary flow rate remained unchanged throughout.

**Conclusions:**

Prolonged gravitational unloading induces a plaque‐driven deterioration of gingival health in systemically healthy individuals, likely related to impaired mechanical plaque control and microcirculatory changes rather than salivary dysfunction. Gingival tissues appear to be sensitive biomarkers of adaptation to unloading conditions, supporting the need for optimized oral hygiene and adjunctive preventive strategies during future long‐duration space missions.

**Trial Registration:**

German Clinical Trial Registry (DRKS00033882, registration date 03‐15‐2024, https://www.drks.de/search/de/trial/DRKS00033882/details).

## 1 Introduction

Human spaceflight induces profound physiological adaptations resulting from exposure to microgravity, including musculoskeletal deconditioning (Greene et al. 2021), cardiovascular adaptations (Tordeur et al. 2025), and neurovestibular disturbances (Hallgren et al. 2016). These systemic changes extend to the craniofacial complex, where headward fluid shift accompanied by immune modulation and altered salivary dynamics may possibly impact oral and periodontal health (Lloro et al. 2020). Despite these potential risks, oral health has historically received limited attention within astronaut health monitoring, even though untreated dental or periodontal conditions can jeopardize mission safety and crew performance (Sadat Kachouei et al. 2025). Microgravity leads to cephalad fluid shifts that alter mucosal hydration, could modify salivary flow, and change the physicochemical environment of the oral cavity (Stevens et al. 2020; Wadhwa et al. 2024). These changes may favor biofilm accumulation, promote gingival inflammation, and potentially shift the salivary and oral microbiome toward a dysbiotic state (Bakr et al. 2024). Immunosuppression and oxidative stress reported in long‐duration missions may exacerbate periodontal tissue susceptibility. Collectively, this highlights that the oral cavity serves as both a sentinel and a mirror of systemic physiological adaptations to microgravity. Beyond physiological alterations, routine oral hygiene practices may also be compromised in spaceflight, as astronauts face technical limitations in performing conventional measures such as toothbrushing, flossing, and rinsing due to microgravity constraints and water conservation requirements. Additionally, dietary changes in space may further influence biofilm accumulation and gingival health. To date, however, no clear recommendations exist regarding optimized oral hygiene protocols or preventive regimens suitable for microgravity environments, representing a critical knowledge gap in aerospace dentistry.

Ground‐based spaceflight analogs, particularly head‐down tilt bed rest (HDTBR), provide a controlled model to study the multisystemic effects of gravitational unloading resulting in cephalad fluid shift (Sun et al. 2025). Previous HDTBR studies have elucidated cardiovascular (Solbiati and Caiani 2025) and neuromuscular (Prokopidis et al. 2025) alterations analogous to those seen postflight; however, systematic assessments of oral health outcomes under these conditions remain scarce. As astronauts in long‐duration missions lack access to in‐flight dental care, understanding the trajectory of periodontal inflammation, bleeding on probing (BoP), biofilm formation, and salivary secretion under simulated microgravity is critical for preventive strategy development.

In the present investigation, we integrated oral health assessments as safety measures into a multidisciplinary 60‐day, 6° HDTBR study. We aimed to document longitudinal changes in oral hygiene status, BoP, pocket depths measured by the Periodontal Screening Index (PSI) and biofilm accumulation, and salivary volume across 60‐day head‐down tilt (HDT) exposure. These parameters were chosen as noninvasive markers of periodontal and mucosal integrity, potentially linked to systemic adaptations under unloading. This approach represents one of the first attempts to contextualize dental safety monitoring within aerospace medicine, bridging the gap between space physiology and periodontology. We expect our findings to enhance understanding of oral‐systemic interactions under gravitational unloading and to inform preventive oral health protocols for future long‐duration missions. Beyond aerospace applications, insights from this analog may also translate to Earth‐based healthy populations with restricted mobility or fluid balance alterations, such as bedridden patients or those with autonomic dysfunction.

## 2 Materials and Methods

### 2.1 Main Study Endpoint and Participant Overview

This study aimed to investigate strategies for preserving sensorimotor function during and after long‐duration space missions. A total of 24 participants were formally enrolled, divided into two experimental campaigns (12 participants per campaign) based on facility availability at the :envihab facility of the German Aerospace Center (DLR), Cologne, Germany. All measurements were conducted as part of the medical care framework of the SMC study. These assessments were not originally designed to address the study's primary objectives but provided relevant complementary data. Accordingly, the present oral health investigation should be interpreted as an exploratory longitudinal sub‐analysis of the SMC study rather than as a primary endpoint‐driven clinical trial.

Besides that, the primary endpoint of the study was the time required to complete the foam obstacle course test, administered before and after HDTBR. Changes in completion time were analyzed to evaluate functional performance and estimate the potential effectiveness of countermeasures applicable to spaceflight operations.

### 2.2 Study Design and Setting

This investigation (reported according to STROBE) was embedded within the Sensorimotor Countermeasures (SMC) Study, a 60‐day HDTBR experiment performed at the :envihab facility. Throughout the bed rest phase, all essential daily activities—including eating, personal hygiene, showering, and bowel movements—as well as leisure activities (e.g., reading or watching television), were performed in the 6° HDT position. Upright postures, such as sitting or standing, were not permitted. Participants were allowed to change body position (supine, prone, or lateral), provided that one shoulder remained in continuous contact with the mattress at all times. The study was designed to simulate the physiological effects of prolonged microgravity exposure, including cephalad body‐fluid redistribution and neuromuscular unloading, under strictly controlled environmental, nutritional, and hygiene conditions (Pavy‐Le Traon et al. 2007). Continuous 24/7 video monitoring ensured adherence to the posture directive and participant safety.

The experimental timeline consisted of three consecutive phases:

1. Baseline data collection (BDC)—15 days for adaptation and reference data acquisition prior HDT (running from BDC‐15 to BDC‐1).
2. HDT phase (running from HDT1 to HDT60)—60 days at a continuous 6° HDT, mimicking cephalad fluid shift and unloading conditions.
3. Recovery phase (R+)—14 days of monitored readaptation to upright posture running from R + 0 to R + 13.

### 2.3 Participants and Recruitment

Healthy volunteers were enrolled following a rigorous multi‐stage screening process comprising comprehensive medical, psychological, and dental evaluations. Eligible participants were confirmed to be physically and psychologically healthy. Only non‐smokers who had abstained from tobacco use for at least 6 months prior to enrollment were accepted. Prior to enrollment, all participants underwent dental clearance within the study eligibility assessment framework and were required to be free of active oral treatment needs at baseline. Specifically, no participant presented with untreated periodontal disease, active caries requiring treatment, acute oral infection, mucosal pathology, or other clinically relevant dental conditions that could pose risk during prolonged immobilization or simulated microgravity exposure. Exclusion criteria encompassed systemic disorders known to affect bone metabolism, inflammatory response, or salivary secretion, as well as recent antibiotic therapy, ongoing orthodontic or periodontal treatment, or the use of medications with potential interference in autonomic or endocrine regulation.

### 2.4 Study Interventions and Groups

The overall SMC study included four HDTBR groups: a head‐down tilt control (CON) group without countermeasures, and three intervention groups undergoing HDT combined with either electrical muscle stimulation (EMS), proprioceptive training (PT), or a combined exercise and PT protocol (EPT) (“countermeasures”). The CON group served to characterize sensorimotor deconditioning in the absence of countermeasures, whereas the EMS, PT, and EPT groups were specifically designed to target muscle activation, proprioceptive and tactile conditioning, and an integrated aerobic‐resistance exercise plus proprioceptive regimen, respectively. All countermeasure interventions were performed in the supine position at 0° tilt, and total time spent in the supine position was matched across all groups to ensure equal exposure.

**Figure 1:**
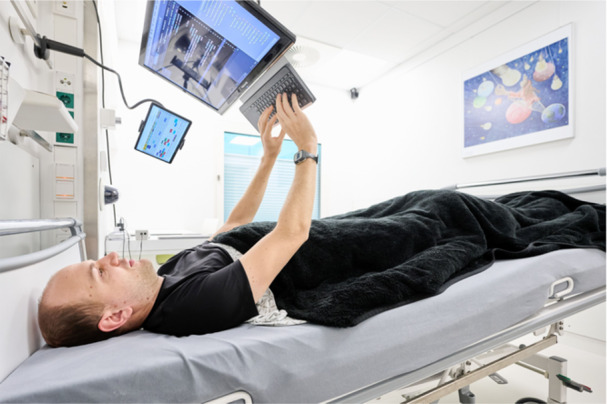
The dental and oral assessments were conducted identically in 6° HDT. Participants remained 60 days at a continuous 6° head‐down tilt. Only a side‐pillow was allowed to assure that the whole body and head remained aligned with the −6° angle.

Dental and oral health assessments as safety measures were uniformly conducted under identical 6° HDT conditions across all groups to identify potential oral or periodontal changes. Importantly, these assessments represented standardized safety measures applied independently of group allocation; thus, dental and oral outcomes were analyzed collectively for the entire study population. Consequently, the results refer to the overall participant cohort rather than to individual intervention groups. Therefore, the dental and oral assessments were conducted identically in 6° HDT (Figure 1), and served as a cross‐cutting safety and exploratory module to detect potential oral changes during prolonged HDT exposure.

### 2.5 Controlled Nutrition and Hygiene Environment

To minimize confounders, participants received a strictly controlled isocaloric diet adjusted for energy requirements and macronutrient balance. Fluid intake and micronutrient content met European Food Safety Authority guidelines. Participants brushed twice daily in the HDT position using individually soft‐bristle toothbrushes (Colgate extra clean medium) and fluoridated toothpaste (Dentagard herbs; 1450 ppm F^−^). No mouth rinses were used. This standardized protocol was implemented to minimize interindividual variability in oral hygiene behavior during the study. Participants who routinely performed interdental cleaning prior to study entry were allowed to continue this practice throughout the study to avoid introducing additional effects related to changes in their habitual oral hygiene routine. Compliance with the hygiene directive was supervised daily by dental staff and recorded using standardized logs.

### 2.6 Dental and Periodontal Assessments

Comprehensive oral examinations were conducted by a single, calibrated investigator (*κ* > 0.8 for BoP and PSI) at four standardized time points: baseline (BDC‐7), mid‐HDT (HDT31), end‐HDT (HDT57), and post‐recovery (R + 6). The periodontal assessment protocol encompassed a structured set of clinical parameters, including BoP (defined as the presence or absence of bleeding within 15 s after gentle probing at six sites per tooth), the modified Quigley‐Hein Plaque Index (QHI) for evaluating supragingival plaque accumulation, and the PSI, with measurements obtained to a precision of 0.2 mm using a UNC‐15 periodontal probe, ensuring reproducibility and compliance with international calibration standards. The German PSI—which corresponds to the Community Periodontal Index of Treatment Needs—was used as a standardized screening tool to monitor clinically relevant periodontal changes during HDT exposure. Given the exploratory nature of the oral health assessments and the pre‐screened periodontal health of the study population at baseline, the primary aim was to detect potential inflammatory deterioration rather than subtle attachment‐level changes. Therefore, PSImax, representing the highest recorded score within an individual's full‐mouth examination, was documented at each time point (BDC‐7, HDT31, HDT57, and R + 6). This index provided a concise categorical classification of periodontal condition, ranging from healthy (Code 0) to severe periodontitis (Code 4), based on pocket depth, calculus presence, and BoP. In addition to objective clinical parameters, subjective oral comfort, hygiene perception, and discomfort were captured using validated visual analog scales (VAS) and verbal rating scales (VRS). All examinations were performed in the horizontal 6° HDT position under standardized illumination, ensuring methodological consistency across all measurement phases.

### 2.7 Saliva Collection

At four examination points (BDC‐10, HDT30, HDT58, and R + 7), unstimulated whole saliva samples were collected to evaluate potential alterations in salivary flow and composition during the HDT phase. Unstimulated saliva was obtained after awakening by passive drooling over a 5‐min period, allowing assessment of basal secretion rates. The collected samples were immediately measured to calculate the unstimulated salivary flow rate (USFR; mL/5 min), providing a quantitative measure of salivary secretion capacity, which represents a sensitive indicator of mucosal hydration and autonomic regulation.

### 2.8 Self‐Reported Parameters

For each examination point, participants completed digital questionnaires (VAS/VRS) reporting information about their oral hygiene, dry mouth symptoms, tooth sensitivity with/without cold stimuli from foods and beverages, changes in taste and smell sensitivity, and bad breath.

### 2.9 Examiner Calibration and Reliability

Two examiners were trained and calibrated on five non‐study volunteers prior to data collection. Inter‐ and intra‐examiner reliability for probing pocket depth remained within ±0.5 mm (*κ* > 0.8).

### 2.10 Statistical Analysis

All data were analyzed using IBM SPSS Statistics version 29.0 (IBM Corp., Armonk, NY, USA). Descriptive statistics are presented as mean ± standard deviation and, where appropriate, median and interquartile range. Continuous outcomes (QHI, GI, BoP, USFR) were analyzed using linear mixed‐effects models. PSImax, as an ordinal variable, was analyzed using Wilcoxon signed‐rank tests with Holm correction. For QHI, GI, BoP, and USFR, time point was included as a fixed effect and participant as a random intercept. This model structure accounts for the non‐independence of observations obtained from the same participant across baseline, HDT, and recovery assessments. The overall fixed effect of time was evaluated using Wald chi‐square tests. Model‐based pairwise contrasts between predefined time points were calculated and adjusted for multiple comparisons within each outcome using the Holm method. Statistical significance was set at *p* < 0.05 (two sided).

## 3 Results

### 3.1 Baseline Characteristics

Twenty‐four systemically healthy participants were included. One participant was excluded after enrollment due to the occurrence of a non‐dental medical finding at an early main examination that necessitated study withdrawal. Therefore, data from 23 participants were analyzed (Table 1). The mean age (range) of the study population was 32.4 ± 6.7 (24–49) years, the mean number of own teeth at presentation was 27.7 ± 1.9, and participants had a moderate number of restorations (mean 4.5 ± 3.8 filled teeth). The sex distribution was balanced (47.8% females, 52.2% males).

**Table 1:** Characteristics of participants (*N* = 23).

Characteristics	Mean ± SD (min–max)
Age	32.4 ± 6.7 (24–49)
No. of teeth	27.7 ± 1.9 (23–32)
No. of teeth with carious lesions	1.2 ± 2.0 (0–7)
No. of filled teeth	4.5 ± 3.8 (0–13)

	*n* (%)
Sex	
Male	12 (52.2)
Female	11 (47.8)
Participants with teeth removed due to orthodontic reasons	
Two first premolars	1 (4.3)
Four first premolars	2 (8.7)
Two third molars	1 (4.3)
Four third molars	18 (78.3)

### 3.2 Oral Hygiene and Periodontal Clinical Findings

All oral hygiene and periodontal clinical findings are outlined in Table 2.

**Table 2:** Changes in objective clinical parameters by examination date (*N* = 23).

	Mean ± SD (min–max)				*p* value*			
	BDC7	HDT31	HDT57	R + 6	BDC7‐HDT31	HDT31‐HDT57	HDT57‐R + 6	BDC7‐R + 6
QHI	1.8 ± 0.4 (1.0–2.4)	2.1 ± 0.4 (1.5–3.2)	2.3 ± 0.4 (1.7–3.0)	2.5 ± 0.4 (1.7–3.3)	**< 0.001**	**0.003**	**< 0.001**	**< 0.001**
GI	0.8 ± 0.4 (0.1–1.5)	1.2 ± 0.2 (0.8–1.2)	1.2 ± 0.2 (0.8–1.2)	1.2 ± 0.2 (0.6–1.7)	**< 0.001**	0.263	0.638	**< 0.001**
BOP (%)	18.4 ± 11.6 (2–45)	28.0 ± 13.2 (9–58)	35.1 ± 16.6 (16–70)	27.7 ± 13.8 (7–58)	**< 0.001**	**< 0.001**	**< 0.001**	**< 0.001**
USFR (mL/5 min)	2.3 ± 1.1 (0.6–5.2)	2.2 ± 1.3 (0.6–6.4)	2.2 ± 1.6 (0.2–6.6)	2.2 ± 2.0 (0.6–9.0)	0.808	0.944	0.834	0.394

	*n* (%)							
PSI max					**0.005**	0.739	0.655	**0.008**
Grade 0	0 (0.0)	0 (0.0)	0 (0.0)	0 (0.0)				
Grade 1	6 (26.1)	2 (8.7)	1 (4.3)	0 (0.0)				
Grade 2	3 (13.0)	2 (8.7)	1 (4.3)	4 (17.4)				
Grade 3	12 (52.2)	13 (56.5)	17 (73.9)	15 (65.2)				
Grade 4	2 (8.7)	6 (26.1)	4 (17.4)	4 (17.4)				

Abbreviations: BDC, baseline data collection; BoP, bleeding on probing; GI, Gingival Index; HDT, head‐down tilt; PSI, Periodontal Screening Index; QHI, Quigley‐Hein Plaque Index; R + , recovery phase; SD, standard deviation; USFR, unstimulated salivary flow rate.

* *p* values for continuous variables are based on linear mixed models with participant as random effect and time as fixed effect; bold values signify statistical significance (*p* < 0.05).

QHI increased progressively from baseline (BDC‐7: 1.83 ± 0.37) to HDT31 (2.13 ± 0.42), HDT57 (2.29 ± 0.42), and R + 6 (2.49 ± 0.42). Model‐based pairwise contrasts showed higher QHI values at HDT31, HDT57, and R + 6 compared with baseline (all Holm‐adjusted *p* < 0.001). QHI further increased from HDT31 to HDT57 (*p* = 0.003) and from HDT57 to R + 6 (*p* < 0.001).

Similarly, GI increased from baseline (BDC‐7: 0.80 ± 0.41) to HDT31 (1.16 ± 0.23), HDT57 (1.23 ± 0.24), and R + 6 (1.21 ± 0.23). Values at all HDT and recovery time points were higher than baseline (all Holm‐adjusted *p* < 0.001), whereas no significant differences were observed between HDT31 and HDT57 or between HDT57 and R + 6. GI values at R + 6 remained elevated indicating persistent gingival inflammation. A positive relationship between plaque accumulation (QHI) and gingival inflammation (GI) was consistently observed throughout the study (Pearson's *r* = 0.11–0.33), although the correlations did not reach statistical significance, likely owing to limited statistical power.

BoP increased from baseline (BDC‐7: 18.37 ± 11.60%) to HDT31 (27.98 ± 13.24%) and HDT57 (35.08 ± 16.60%). BoP decreased at R + 6 (27.69 ± 13.80%) compared with HDT57 (Holm‐adjusted *p* < 0.001) but remained higher than baseline (Holm‐adjusted *p* < 0.001).

A significant shift toward higher PSI grades was observed between baseline and HDT31 (*p* = 0.005) and between baseline and R + 6 (*p* = 0.008). While PSI Grade 3 predominated at all time points, the proportion of participants presenting with PSI Grade 4 increased during the experimental phase and remained elevated at R + 6. No changes in PSI distribution were detected between HDT31 and HDT57 or between HDT57 and R + 6 (*p* > 0.05).

USFR remained stable throughout the study, with mean values of 2.26 ± 1.13 mL/5 min at BDC‐10, 2.14 ± 1.28 mL/5 min at HDT30, 2.16 ± 1.60 mL/5 min at HDT58, and 2.23 ± 1.97 mL/5 min at R + 7. Neither the overall time effect nor any model‐based pairwise comparison indicated significant changes in USFR (Table 2).

In conclusion, linear mixed‐effects models demonstrated significant longitudinal changes in QHI, GI, and BoP (all *p* < 0.001), whereas no significant effect of time was observed for USFR (*p* = 0.987).

### 3.3 Self‐Reported Parameters

Standardization of oral hygiene to a manual toothbrush did not affect brushing time over the study duration (all *p* > 0.05, Table 4). Approximately one‐third of participants reported a reduction in the subjective cleanliness of their teeth during the HDT phase and at R + 6 (Table 4). The perception of bad breath/taste increased up to HDT31 (*p* < 0.001) and remained stable thereafter (Table 4). Self‐reported tooth sensitivity, both overall and in response to cold food or beverages, decreased significantly from BDC7 to HDT31 according to the VAS assessments (both *p* < 0.001) and remained low throughout the remainder of the study period (Table 3). Corresponding VRS measures showed the same directional trend but did not reach statistical significance after correction for multiple testing (Table 3). Only a small number of participants reported subjective changes in taste or smell, most commonly describing a reduction in these sensory perceptions. Owing to the high proportion of missing responses after baseline, these data are presented descriptively only (Table 4).

**Table 3:** Changes in self‐reported oral health parameters by examination date (*N* = 23).

	VAS, n mean ± SD (min–max)				*p* value*			
	BDC7	HDT31	HDT57	R + 6	BDC7‐HDT31	HDT31‐HDT57	HDT57‐R + 6	BDC7‐R + 6
Dry mouth symptoms (%)	9 17.1 ± 23.3 (1–76)	7 30.1 ± 10.5 (15–43)	8 24.3 ± 12.4 (5–47)	3 27.7 ± 36.7 (4–70)	0.684	1.000	1.000	1.000
Tooth sensitivity (%)	23 18.4 ± 19.4 (0–67)	23 6.6 ± 10.3 (0–33)	23 5.3 ± 9.0 (0–26)	23 5.2 ± 8.4 (0–24)	**< 0.001**	1.000	1.000	**< 0.001**
Tooth sensitivity due to cold food or beverages (%)	23 15.0 ± 14.8 (0–51)	23 5.3 ± 8.6 (0–29)	23 4.0 ± 7.5 (0–27)	23 4.1 ± 5.7 (0–18)	**< 0.001**	1.000	1.000	**< 0.001**

	VRS, *n* (%) (*N* = 23)							
Tooth sensitivity					0.081	0.858	0.858	0.098
No	8 (34.8)	17 (73.9)	15 (65.2)	15 (65.2)				
Slight	14 (60.9)	5 (21.7)	6 (26.1)	8 (34.8)				
Moderate	1 (4.3)	1 (4.3)	2 (8.7)	0 (0.0)				
Severe	0 (0.0)	0 (0.0)	0 (0.0)	0 (0.0)				
Extreme	0 (0.0)	0 (0.0)	0 (0.0)	0 (0.0)				
Tooth sensitivity due to cold food/beverages					0.098	1.000	1.000	0.081
No	11 (47.8)	18 (78.3)	18 (78.3)	19 (82.6)				
Slight	11 (47.8)	5 (21.7)	5 (21.7)	4 (17.4)				
Moderate	1 (4.3)	0 (0.0)	0 (0.0)	0 (0.0)				
Severe	0 (0.0)	0 (0.0)	0 (0.0)	0 (0.0)				
Extreme	0 (0.0)	0 (0.0)	0 (0.0)	0 (0.0)				

Abbreviations: BDC, baseline data collection; HDT, head‐down tilt; R+, recovery phase; SD, standard deviation; VAS, visual analog scale; VRS, verbal rating scale.

* *p* values for continuous VAS outcomes are based on linear mixed‐effects models with participant as random effect and time as fixed effect. Pairwise comparisons were adjusted using the Holm method. *p* values for ordinal VRS outcomes are based on Wilcoxon signed‐rank tests with Holm correction. Bold values indicate statistical significance (*p* < 0.05).

**Table 4:** Changes in self‐reported categorical oral health parameters by examination date (*N* = 23).

		*n* (%)				*p* value^a^			
		BDC7	HDT31	HDT57	R + 6	BDC7‐HDT31	HDT31‐HDT57	HDT57‐R + 6	BDC7‐R + 6
Duration of tooth brushing						1.000	1.000	1.000	1.000
About 1 min		1 (4.3)	1 (4.3)	2 (8.7)	1 (4.3)				
About 2 min		9 (39.1)	9 (39.1)	7 (30.4)	10 (43.5)				
About 3 min		9 (39.1)	12 (52.2)	12 (52.2)	10 (43.5)				
> 3 min		3 (13.0)	0 (0.0)	2 (8.7)	2 (8.7)				
It differs		1 (4.3)	1 (4.3)	0 (0.0)	0 (0.0)				
Change in subjective cleanliness of teeth due to standardization of toothbrush and toothpaste							0.635	0.472	
Yes		—	9 (39.1)	7 (30.4)	9 (39.1)				
No		—	14 (60.9)	16 (69.6)	14 (60.9)				
Reduced		—	7 (77.8)	7 (100.0)	8 (34.8)				
Better		—	0 (0.0)	0 (0.0)	1 (4.3)				
N/A		—	2 (22.2)	0 (0.0)	0 (0.0)				
Bad breath/taste						**< 0.001**	0.366	0.140	**< 0.001**
Daily		1 (4.3)	0 (0.0)	2 (8.7)	3 (13.0)				
Once a week		1 (4.3)	5 (21.7)	3 (13.0)	3 (13.0)				
Several times a week		1 (4.3)	6 (26.1)	3 (13.0)	4 (17.4)				
Several times a month		8 (34.7)	8 (34.7)	10 (43.5)	10 (43.5)				
Never		7 (30.4)	0 (0.0)	0 (0.0)	0 (0.0)				
It differs		2 (8.7)	0 (0.0)	0 (0.0)	0 (0.0)				
Don't know		3 (13.0)	4 (17.4)	5 (21.7)	3 (13.0)				
Sense of taste									
Normal		23 (100.0)	9 (39.1)	9 (39.1)	9 (39.1)				
Reduced (hypogeusia)		0 (0.0)	1 (4.3)	2 (8.7)	1 (4.3)				
No taste (ageusia)		0 (0.0)	1 (4.3)	0 (0.0)	1 (4.3)				
Missing		0 (0.0)	12 (52.2)	12 (52.2)	12 (52.2)				
Sense of smell									
Normal		23 (100.0)	9 (39.1)	7 (30.4)	7 (30.4)				
Reduced		0 (0.0)	2 (8.7)	1 (4.3)	3 (13.0)				
Increased		0 (0.0)	0 (0.0)	2 (8.7)	0 (0.0)				
Missing		0 (0.0)	12 (52.2)	12 (52.2)	13 (56.5)				

Abbreviations: BDC, baseline data collection; HDT, head‐down tilt; N/A, not applicable; R+, recovery phase.

^a^ Overall changes in ordinal outcomes were evaluated using Friedman tests. Pairwise comparisons were performed using Wilcoxon signed‐rank tests with Holm correction for multiple testing. Bold values indicate statistical significance (*p* < 0.05). Taste and smell variables are reported descriptively because of the high proportion of missing responses after baseline.

## 4 Discussion

We investigated longitudinal changes in oral hygiene and periodontal health during a 60‐day HDTBR study as a validated ground‐based analog of microgravity exposure (Watenpaugh 2016). The baseline characteristics describe a systemically healthy, young to middle‐aged cohort with generally good oral health, providing an appropriate model to detect subtle but clinically relevant periodontal responses to HDT. Consistent findings across clinical parameters demonstrated a measurable deterioration of gingival and periodontal conditions under HDT, indicating that microgravity analog environments can disrupt oral homeostasis even in otherwise healthy individuals.

The progressive increase in plaque accumulation and BoP during HDT reflects a shift toward a pro‐inflammatory gingival environment. GI values remained elevated during recovery, suggesting ongoing inflammatory adaptation even after unloading ceased. In contrast, plaque accumulation continued to increase after HDT. Two interrelated mechanisms likely contributed to these findings. First, cephalad fluid shift inherent to the HDT model may increase venous pressure and tissue congestion in the head and neck region (Sun et al. 2025), resulting in subclinical gingival edema and increased bleeding susceptibility. Second, the HDT position imposes functional limitations on oral hygiene performance, facilitating plaque retention due to restricted mobility, altered hand‐eye coordination, and the supine orientation—conditions comparable to those encountered during spaceflight (Sadat Kachouei et al. 2025). Together, impaired biofilm control and altered microcirculatory dynamics provide a plausible explanation for the observed increases in plaque, gingival inflammation, bleeding, and the shift toward higher PSI grades. The increase in Grade 4 scores observed during HDT and persisting into recovery suggests an inflammatory burden rather than established attachment loss, supporting the interpretation of a predominantly inflammatory, gingivitis‐like phenotype. This pattern is consistent with previous spaceflight and immobilization studies reporting mucosal swelling, altered capillary pressure, and immune modulation associated with headward fluid shifts (Hargens et al. 1994; Ly et al. 2022). Nevertheless, even pathologic increased pocket depths provide the ecologic environment for a more disease‐related biofilm composition, and a significant decrease in bone and mineral density has previously been reported.

The objective impairment in biofilm control was accompanied by a self‐perceived worsening of tooth cleanliness in one‐third of participants. Additionally, self‐reported bad breath/taste increased in the HDT‐phase and was prolonged after unloading, which is consistent with microbial changes in gingivitis and periodontitis (Pham et al. 2012) and in accordance with the increasing PSI scores.

Self‐reported tooth‐sensitivity, with or without stimuli from cold food or beverages, decreased in the HDT‐phase and remained low after unloading was evaluated. This may be attributed to fluid shift to the head, which also impacts the amount of fluid in the dentinal tubules—thus reducing the strength of fluid movement and desensitizing the overall odontoblast processes and to thermal sensations such as cold (Brännström 1986).

Unstimulated salivary flow remained stable throughout the study, indicating that quantitative salivary secretion was not impaired by gravitational unloading. Thus, the observed periodontal changes were likely driven by local plaque accumulation and vascular tissue responses rather than hyposalivation. Nevertheless, potential qualitative changes in salivary composition cannot be excluded and may have contributed indirectly to inflammatory susceptibility. Even though the USFR remained constant, some subjects reported subjective dry mouth symptoms, the severity of which did not differ between appointments—although the variance during the HDT phase was lower, which may be attributed to the strict food and liquid intake.

Compared with ground‐based cohorts maintaining an upright posture, who typically show stable plaque and gingival indices under routine hygiene conditions (Lei et al. 2025), the deterioration observed in our study underscores the intrinsic influence of body position and gravitational unloading. Our findings further support the concept that gingival and periodontal parameters may serve as sensitive biomarkers of systemic and hemodynamic adaptation to altered gravity.

From an operational and preventive perspective, the results have important implications for human spaceflight. During long‐duration missions, astronauts face inherent constraints in mechanical oral hygiene, altered microcirculation (Barravecchia and Angeloni 2022), and lack of professional dental care. Even mild gingival inflammation or bleeding may impair comfort and operational performance, particularly under conditions of microgravity‐associated immune dysregulation (Pelchat et al. 2025). Given the predominantly plaque‐driven and partially reversible nature of the observed changes, optimization of oral hygiene prior to mission launch and adjunctive chemical plaque control strategies may represent feasible preventive countermeasures. In addition to reinforced oral hygiene instruction, antiseptic or anti‐inflammatory agents—such as chlorhexidine‐containing gels, or essential‐oil formulations—may help reduce inflammatory burden when optimal mechanical plaque removal cannot be maintained. Furthermore, water‐independent oral hygiene systems and compact interdental cleaning devices may provide practical alternatives under microgravity conditions where conventional hygiene procedures are operationally limited. Importantly, the incomplete normalization of several periodontal parameters during recovery suggests that post‐mission dental monitoring and supportive preventive care may also be necessary following long‐duration unloading exposure. Future studies should evaluate the efficacy, tolerability, and operational feasibility of such preventive and post‐mission oral health strategies under simulated and real microgravity conditions.

Our study is limited by its small sample size and preliminary nature, which may have reduced statistical power to detect subtle effects. This applies, for example, to the association between plaque accumulation and gingival inflammation. Positive correlations were consistently observed throughout the study (Pearson's *r* = 0.11–0.33), supporting the biologically plausible relationship between increasing plaque accumulation and gingival inflammation. Although the magnitude of the associations reached a moderate level during HDT exposure, statistical significance was not achieved, most likely because the study was powered for the primary sensorimotor endpoint of the NASA SMC study rather than for exploratory dental correlation analyses. While the HDT model effectively reproduces cephalad fluid shifts (Sun et al. 2025), it does not account for additional spaceflight stressors such as radiation, confinement stress, or true microgravity biomechanics. The absence of microbiological, inflammatory, and salivary biomarker analyses further limits mechanistic insight into the observed gingival changes. Consequently, the proposed mechanisms involving cephalad fluid shifts, altered plaque control, and inflammatory adaptation should be interpreted as biologically plausible explanations rather than directly demonstrated causal pathways. Furthermore, periodontal evaluation was intentionally based on a screening‐oriented approach using PSI rather than comprehensive periodontal charting with full‐mouth probing depth and clinical attachment level measurements. As participants had undergone extensive dental pre‐screening and were charted as periodontal healthy, the objective was to identify clinically relevant inflammatory deterioration under unloading conditions rather than subtle periodontal tissue changes. Furthermore, the standardization of oral hygiene procedures, and toothbrushing under continuous HDT conditions, may itself have contributed to impaired plaque removal and gingival inflammation. However, this represents a clinically relevant aspect of the unloading model, as similar operational limitations in performing routine oral hygiene may occur during spaceflight. Nevertheless, the controlled environment, repeated‐measures design, and integration of clinical and salivary assessments provide valuable insight into oral physiological responses under one of the most valid Earth‐based microgravity analogs.

## 5 Conclusions

Prolonged gravitational unloading during HDTBR induces a deterioration with only partial recovery during the observed recovery phase, primarily driven by impaired plaque control and microcirculatory fluid shifts rather than salivary dysfunction or structural periodontal breakdown. Gingival tissues appear to be sensitive biomarkers of systemic adaptation to unloading conditions. For space missions with a duration of approximately 60 days, clinically significant periodontal disease is unlikely if preventive standards are maintained. However, even minor inflammatory changes may carry operational relevance. These risks may be mitigated by pre‐mission optimization of oral hygiene and, where appropriate, providing adjunctive anti‐inflammatory or antiseptic oral care products. Accordingly, the principle of “no oral emergencies in space” should remain central to crew medical certification and preventive countermeasure development.

## Author Contributions

**Sonja Henny Maria Derman:** conceptualization, methodology, scientific supervision of data collection, data curation, formal analysis, statistical analysis, scientific advice on study endpoints. **Laura de Boni:** investigation, project administration, quality assurance of study procedures, participant recruitment and supervision during the study phase. **Edwin Mulder:** investigation, project administration, quality control and implementation of study procedures, participant supervision during the study phase. **Stefan Möstl:** project administration, quality control and implementation of study procedures, participant supervision during the study phase. **Julia Jagel:** data collection, contribution to manuscript preparation. **Teresa Kruse:** methodology, scientific advice and implementation regarding study endpoints. A**nna Greta Barbe:** writing – original draft, writing – review and editing, data interpretation, supervision, validation of dental data and overall scientific quality assurance. All authors reviewed and approved the final version of the manuscript.

## Funding

The authors have nothing to report.

## Ethics Statement

Ethics committees in Germany (Ärztekammer Nordrhein, 2023289) and in the United States (IRB at Johnson Space Center, STUDY00000661) approved the study. Approval to apply ionizing radiation was obtained from the German Federal Office for Radiation Protection (Bundesamt für Strahlenschutz, ZD3—22464/2024‐071‐G). All participants gave their written informed consent.

## Conflicts of Interest

The authors declare no conflicts of interest.

## Data Availability

The data that support the findings of this study are available from the corresponding author upon reasonable request.
